# Comparison of Anticancer Drug Toxicities: Paradigm Shift in Adverse Effect Profile

**DOI:** 10.3390/life12010048

**Published:** 2021-12-29

**Authors:** Debasish Basak, Scott Arrighi, Yasenya Darwiche, Subrata Deb

**Affiliations:** Department of Pharmaceutical Sciences, College of Pharmacy, Larkin University, Miami, FL 33169, USA; DBasak@ULarkin.org (D.B.); SArrighi@myularkin.org (S.A.); YDarwiche@myularkin.org (Y.D.)

**Keywords:** adverse effects, antibody, cytochrome P450, large molecules, small molecules, toxicities

## Abstract

The inception of cancer treatment with chemotherapeutics began in the 1940s with nitrogen mustards that were initially employed as weapons in World War II. Since then, treatment options for different malignancies have evolved over the period of last seventy years. Until the late 1990s, all the chemotherapeutic agents were small molecule chemicals with a highly nonspecific and severe toxicity spectrum. With the landmark approval of rituximab in 1997, a new horizon has opened up for numerous therapeutic antibodies in solid and hematological cancers. Although this transition to large molecules improved the survival and quality of life of cancer patients, this has also coincided with the change in adverse effect patterns. Typically, the anticancer agents are fraught with multifarious adverse effects that negatively impact different organs of cancer patients, which ultimately aggravate their sufferings. In contrast to the small molecules, anticancer antibodies are more targeted toward cancer signaling pathways and exhibit fewer side effects than traditional small molecule chemotherapy treatments. Nevertheless, the interference with the immune system triggers serious inflammation- and infection-related adverse effects. The differences in drug disposition and interaction with human basal pathways contribute to this paradigm shift in adverse effect profile. It is critical that healthcare team members gain a thorough insight of the adverse effect differences between the agents discovered during the last twenty-five years and before. In this review, we summarized the general mechanisms and adverse effects of small and large molecule anticancer drugs that would further our understanding on the toxicity patterns of chemotherapeutic regimens.

## 1. Introduction

The term “cancer” is usually associated with negative memories, hardships, and challenges by the society despite the progress that has been made over the past decades in treating different types of malignancies. According to the World Health Organization (WHO), approximately 10 million deaths were reported in 2020 from this debilitating disease [1]. The National Cancer Institute (NCI) defines cancer as a term for diseases in which abnormal cells divide without control and can invade nearby tissues (www.cancer.gov, accessed on 12 June 2021). The term “chemotherapy” is also commonly misunderstood. Chemotherapy simply means using a chemical as a therapy, rather than total association with cancer treatment. Oncological drugs are known for their potent adverse effects. It is important to comprehend these toxicities/adverse effects and why they are caused to prevent them from happening. Broadly, toxicity refers to the extent to which something is detrimental. Most of these drugs have very narrow therapeutic windows, making it difficult to render an effective therapeutic dose without experiencing toxic effects [2].

Earlier anticancer medications have more general mechanisms of actions, which narrow the therapeutic windows even further. These drugs are usually small molecules between 200 and 500 Daltons. The newer agents are entirely proteins and therefore are much larger, being around 150 kilodaltons. They target their sites of actions more specifically, carry less side effects, and are mostly administered parenterally and have stark differences in pharmacokinetics with small molecules. In terms of metabolism, small molecules are usually metabolized by cytochrome P450 (CYP) enzymes or conjugated, while large molecules are usually broken down catabolically into peptides or amino acids. When observing drug–drug interactions, most of the small molecules and their interactions have been identified and thoroughly studied. Large molecules, also known as biologics, are relatively new and consequently less studied. The development of drugs that treat cancer has been a long process in history veering toward larger molecules.

The newer and larger biologic agents represent a brand-new era for medicine and particularly for oncology. With cancer medications relying largely on cytotoxicity for benefit, it is encouraging to see that more targeted medications are becoming available. These medications remain effective at killing the malignant cells but are devoid of the same level of toxicity. Thee biologics can trigger a host immune system causing immunogenicity issues [3] and is usually not a problem for the small molecules. The small molecule agents seem to have much greater variation in their bioavailability due to environmental or genetic factors. These factors affect the concentration of small molecules much more than large molecules because small molecules depend primarily on CYP enzymes and other ADME actions. However, there are new challenges, since almost all the newer agents have a black box warning or a greater chance of causing an infusion reaction. This has led to polypharmacy with the prophylactic treatment for infusion reactions with medications including H1 and H2 receptor antagonists, corticosteroids, and antiemetics. There are also newer delivery systems for older medications. Similar to many small oncological drug molecules, paclitaxel has an issue with protein binding. This can lead to an enormous variability of effect as well as change the effect of other medications. There is now an albumin bound paclitaxel that lowers this variability, resulting in better outcome for the patient [4]. However, the major issue with all these innovations is the cost.

Apart from surgery and radiation, chemotherapy is the most common approach in the treatment of cancer. Although the complete cure with chemotherapy results roughly in about 5% of cancer patients, it is commonly administered to a significant number of patients with the appearance of myriad unwanted/adverse effects. These toxicities arise due to the resistance of tumor cells to chemotherapy and because of the similarity between cancer cells with normal cells in terms of structure and function [5]. The pattern of toxicities between small and large molecules is of huge research interest. This review offers an overview of diverse toxicities of these anticancer drug molecules, and a better understanding of these toxicities may facilitate future treatment decision in cancer patients.

## 2. History of Cancer Chemotherapy Drugs

To compare the toxicities of the chemotherapeutics, it is important to recognize the chronological progress in this field. In 1941, Charles Huggins began the process of hormonal therapy by removing testicles of men and administered estrogens to help reduce the progression of prostate tumors [6]. A few years down the line in 1947, the first antimetabolites were used to help children with acute leukemia to go into temporary remission. Two years later, the first alkylating agent was used to treat cancer [7]. Next, in 1958, doctors used a combination therapy of 6-mercaptopurine and methotrexate to achieve partial and complete remissions in the treatment of both children and adults with acute leukemia. This was evident that administering more than one agent with diverse mechanisms could be helpful to overcome the resistance and extend remission. However, adverse events became more prominent since the use of combination therapy. After a year in 1959, cyclophosphamide exhibited promising results in lymphoid leukemia with less severe thrombocytopenia [8]. In 1962, vincristine was introduced, and it provided complete remissions of childhood lymphoid leukemia that was resistant to other agents. However, akin to earlier agents, these remissions were temporary, and a more advanced, drug-resistant leukemia often occurred [9]. In 1978, tamoxifen, a medication originally used as birth control, was approved in treating breast cancer. Furthering the development of breast cancer treatment, the first aromatase inhibitor, Anastrozole, was approved in 1996 [8].

Rituximab, the first large molecule for cancer therapy, was introduced in the 1990s. At that time, it was indicated for non-Hodgkin lymphoma but later was used for a variety of cancers [10]. Moving forward in 1998, trastuzumab was introduced in the market to target the Human Epidermal Growth Factor Receptor (HER2) that is commonly expressed on the surface of breast cancer cells [11]. In 2001, imatinib was developed to treat chronic myelogenous leukemia [8]. A decade later in 2011, ipilimumab, a monoclonal antibody, was approved for metastatic melanoma. This event was followed by the approval of pembrolizumab in 2014, and this agent was also used for melanoma [12]. As observed, the timeline of chemotherapeutic drug innovation has long been improving and continues to today. The classification of oncologic drugs is idiosyncratic and should be analyzed for toxicity independently. Table 1 highlights the timeline of key anticancer treatment.

## 3. Highlights of Chemotherapeutic Drugs and Their Adverse Drug Effects

**Figure 1:**
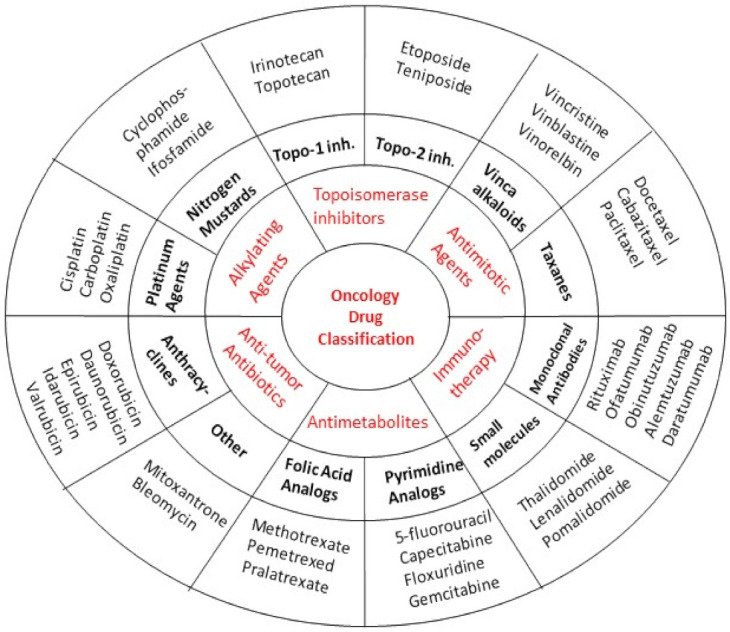
Classification of major anticancer drugs along with their subclasses. This is a representative classification based on mechanism of action. The red letters indicate the major anticancer drug classes, and the black bold letters represent the corresponding examples of drug subclasses.

Practically, no anticancer agent is devoid of toxicity. Depending on their mechanism of action, they are categorized into different classes and subclasses. This section of our review discusses the adverse effects that are caused by the commonly used anticancer drugs. Figure 1 illustrates the major anticancer drug classes along with the representative agents in terms of their general mechanisms of action, and Table 2 displays the prototype adverse effect(s) of these agents.

### 3.1. Antimetabolites

Antimetabolites are one of the oldest drug classes in oncology, and all the agents under this class are small molecules. Methotrexate is a prototype folic acid analog in this class, and one of its major adverse reactions is acute renal failure that can be exacerbated by a nephrotoxic agent, cisplatin. The concomitant use of methotrexate with other renal-impairing agents may delay its excretion, which may result in severe myelosuppression [38]. Methotrexate gets precipitated into the renal tubules that result in nephrotoxicity [39]. Neurotoxicity is another potential side effect that develops mainly due to higher dosing and when administered intravenously [40]. Hepatotoxicity that is commonly seen in renally impaired patients occurs due to the inhibition of DNA and RNA synthesis in the liver [41]. 5-fluorouracil (5-FU) is a prime example of the pyrimidine analog that exerts several adverse effects including gastrointestinal (GI) issues, leukopenia, thrombocytopenia, and hemorrhage. However, these toxicities depend on the route of administration and duration of treatment [42].

### 3.2. Alkylating Agents

This anticancer drug class also encompasses the small molecules. Cyclophosphamide is a nitrogen mustard-type alkylating agent that is associated with myelosuppression as well as neutropenia, anemia, and thrombocytopenia. The reported mechanism of myelosuppression from cyclophosphamide is related to Nrf2 deficiency that resulted in diminished antioxidant capacity and drug detoxification [43]. This is important to note that based on the duration of treatment, cyclophosphamide can cause infertility because of the inability of the cells to differentiate and replicate [44]. Cisplatin is a prominent platinum-based agent that carries black box warnings on myelosuppression and nephrotoxicity. The nephrotoxicity is caused by the accumulation of the drug through organic cation transporter 2 and the activation of several pathways such as MAP kinase, p53, and DNase 1 pathways. Moreover, cisplatin may trigger permanent ototoxicity [45]. The cause of this severe toxicity is not well understood, but the dose-based relationship is evident. Cisplatin also has a black box warning for nausea and vomiting that may be prevented with the use of anti-emetic regimens [46,47]. Carboplatin, another platinum-based agent, usually produces myelosuppression that is characterized by thrombocytopenia, granulocytopenia, and anemia [48]. Oxaliplatin results in peripheral neuropathy and mild to moderate vascular pain at the site of injection. The exact reason of vascular pain is not clear; however, peripheral neuropathy occurs due to the destruction of sensory neurons through the binding of the drug to the sensory neuron DNA [49].

### 3.3. Antitumor Antibiotics

#### 3.3.1. Anthracyclines: Doxorubicin

Antitumor antibiotics represent a very important class of old and small molecule chemotherapeutics, and doxorubicin is a prototypical example of anthracycline subclass. A trademark toxicity of doxorubicin is cardiomyopathy that is caused by the oxygen free radicals and lipid peroxidation within the myocardium [50]. Another unique adverse effect of doxorubicin is DNA damage-induced secondary leukemia, which is also a black box warning [51]. Moreover, doxorubicin can also produce myelosuppression and blood vessel damage through extravasation [52].

#### 3.3.2. Topoisomerase I Inhibitor: Camptothecins

Camptothecin, a small molecule topoisomerase I inhibitor, causes DNA damage by generating torsional strain on DNA. One of the commonly used camptothecins is irinotecan that has hallmark black box warnings of diarrhea and myelosuppression [53]. Irinotecan is a prodrug that is cleaved by carboxylesterase to release the active lipophilic metabolite, 7-ethyl-10-hydroxycamptothecin (SN-38). Mechanistically, bacterial ß-glucuronidase deconjugates SN38-glucuronide (SN38G) to the active form SN38, leading to diarrhea [54]. 

#### 3.3.3. Anthracenedione: Mitoxantrone

Mitoxantrone, the most common small molecule anthracenedione, causes less cardiomyopathy by virtue of not forming quinone-type free radicals. The black box warnings of mitoxantrone include extravasation, neutropenia, and secondary leukemia. Other side effects include GI and menstrual disorders and the blue discoloration of the urine, which are dose-related and reversible [55].

### 3.4. Antimitotic Agents

#### 3.4.1. Vinca Alkaloids

Currently, four major vinca alkaloids are used in practice, and all these are small molecules: vincristine, vinblastine, vindesine, and vinorelbine. Among these, vincristine needs to be used intravenously, and some of its major adverse reactions comprise of leukopenia, thrombocytopenia, and anemia due to the inability of the cells to replicate. Neurological adverse effects such as peripheral neuropathy are more commonly seen with this drug than other cancer drugs. Vincristine deregulates axonal transport and damages nerve axons after binding to microtubules, thereby inducing neuronal death [56]. Since vinca alkaloids are heavily metabolized by the CYP3A isoforms in the liver, concomitant CYP3A inducers and inhibitors should be closely monitored or avoided; otherwise, they can result in either increased toxicity or decreased efficacy of the drugs [57].

#### 3.4.2. Taxanes

The taxanes class consists of small molecules, and paclitaxel is the most common taxane that carries a black box warning for anaphylaxis because of the infusion, and therefore, patients should be pretreated with histamines, corticosteroids, and H_2_ antagonists. Other adverse effects include neutropenia and peripheral neuropathy [58]. Two other commonly used taxanes are docetaxel and cabazitaxel. While docetaxel-treated patients frequently exhibit febrile neutropenia, infusion reactions, and fluid retention, cabazitaxel treatment is associated mostly with hematological adverse effects such as neutropenia, leukopenia, and anemia [59,60]. Cabazitaxel has black box warnings for neutropenia and hypersensitivity reactions [60]. Paclitaxel and docetaxel are solubilized in cremophor and polysorbate 80 solvents, respectively, and these solvents can generate anaphylatoxins C3a and C5a that are associated with anaphylaxis [61].

### 3.5. Hormonal Agents

#### 3.5.1. Androgen Receptor Antagonists

The newest class of medications used exclusively for prostate cancer is the androgen receptor antagonists that inhibit the binding of testosterone and dihydrotestosterone (DHT) with androgen receptors [62]. An example of this class is a small molecule enzalutamide that produces some common adverse reactions such as fatigue, muscular pain, and headache [63]. The fatigue and muscle pain could be due to the medication not being as specific to the prostate as was previously thought. Other side effects include decreased appetite and hot flushes, which are common among hormonal therapies [62].

#### 3.5.2. CYP17A Inhibitors

CYP17 inhibitors are another widely used drug class in the treatment of prostate cancer. The small molecule abiraterone is the most common drug in this category, and hypertension and hypokalemia are hallmark side effects of this agent. Other side effects include increased triglycerides and liver enzymes [64]. Hypertension arises because of a decrease in androgen synthesis and an elevated mineral corticoid production, both of which are linked to CYP17A1 inhibition [65]. 

#### 3.5.3. Selective Estrogen Receptor Modulators (SERMS)

SERMS that target female hormones are used as breast cancer medications, and the agents in this drug class are small molecules. Tamoxifen is a common SERM that comes with a black box warning for uterine malignancies, stroke, and pulmonary embolism. This is because it mimics estrogen in certain tissues that increases the risk of blood clots and endometrial cancer. Possible side effects due to the hormonal changes comprise of hot flushes, vaginal discharge, changes in the lining of the endometrium, and blood clots [66].

#### 3.5.4. Selective Estrogen-Receptor Down Regulators (SERDS)

SERDS are another class of breast cancer drugs. Fulvestrant is the most well-known small molecule in this class, and its most clinically significant adverse reactions are due to loss of estrogenic effects, leading to bone pain, hot flushes, nausea, headache, and anorexia [66].

#### 3.5.5. Aromatase Inhibitors

Aromatase inhibitors are the small molecule hormonal agents that block the aromatase enzyme, which is responsible for converting androstenedione and testosterone into estrone and estradiol, respectively. Anastrozole, a non-steroidal aromatase inhibitor, is not associated with any black box warning; however, it can still demonstrate a few adverse reactions such as arthritis, arthralgia, and back pain [67]. These reactions are due to a lack of estrogen production and hormonal imbalance. When compared with other hormonal agents, anastrozole is associated with a higher incident of musculoskeletal disorders and fractures [68].

### 3.6. Kinase and Signal Transduction Antagonists

#### 3.6.1. EGFR Antagonists

##### EGFR (ErbB1) Blocker- Cetuximab

The kinase and signal transduction antagonists that modulate the signaling proteins and block their action are newer and larger molecules. EGFR (epidermal growth factor receptor) antagonists are a subclass in this category. Cetuximab is a common biologic that has the typical black box warnings of infusion reaction and cardiopulmonary arrest. Dermatologic reaction associated with acne is another common toxicity associated with cetuximab [69]. This drug promotes an increased expression of cytokines that mediate the dermal toxicity [70].

##### HER2 Blocker: Trastuzumab

HER2 is a protein that is overexpressed in breast cancer [71]. Trastuzumab, a biological large molecule, is an HER2 blocker that carries the black box warning of cardiomyopathy, especially in patients who are concomitantly on anthracycline-containing chemo-regimens [72]. This causes a decrease in the left ventricular ejection fraction. Similar to most infused drugs, there is a black box warning for infusion reaction, although trastuzumab’s infusion correlated with pulmonary toxicity [73]. Trastuzumab elicits cardiotoxicity by deregulating HER signaling and activating autophagy-inhibitory Erk/mTOR/Ulk 1 signaling cascade. This results in the inability of cardiomyocyte to recycle toxic cellular substrates, leading to cardiotoxicity [72].

##### Tyrosine Kinase Inhibitors

Tyrosine kinase inhibitors are small molecule medications and therefore can get inside the cell to inhibit tyrosine kinase. Gefitinib is an important drug in this class that furnishes mostly mild adverse reactions including a high frequency (>40%) of both diarrhea and rash, while also displaying a lower frequency of nausea, vomiting, dry skin, and acne [74].

#### 3.6.2. BCR-ABL Inhibitors

The small molecule drugs in this class such as imatinib works by inhibiting the BCR-ABL tyrosine kinase that is created by the Philadelphia chromosome fusion gene mutation [75]. When taking this medication, patients can experience edema, rash, musculoskeletal pain, hepatotoxicity, nausea, and vomiting. While hepatotoxicity is related to the dose and is affected by CYP mediated reactions, the other adverse reactions are associated with discontinuation of the drug. Imatinib promotes reactive oxygen species (ROS) formation, damages mitochondria, and reduces glutathione pool in the liver and mediates hepatotoxicity [76]. Other agents in this class are dasatinib, nilotinib, and bosutinib. The warnings associated with these agents include bleeding events, QT prolongation, and congestive heart failure [77].

#### 3.6.3. RAS/MAP Kinase Pathway

Another class of drugs interfering with the kinase pathway include RAF inhibitors. Sorafenib, a representative small molecule drug in this class, demonstrates a few adverse reactions that include bleeding, dermatologic events, diarrhea, and hypertension [78].

#### 3.6.4. CDK4/6 Inhibitor

The next class comprises of the CDK4/6 inhibitors that include palbociclib, ribociclib, and abemaciclib, all of which are small molecules. Their common adverse reactions are neutropenia, leukopenia, and infections. Neutropenia is the primary area of concern that is seen in most patients and should be monitored throughout the treatment. These effects are a direct cause of not being able to produce white blood cells due to the reduction in DNA replication [79]. Stomatitis is another side effect seen commonly in patients taking palbociclib and should be monitored for tolerance [80].

### 3.7. Angiogenesis Inhibitors

#### 3.7.1. VEGF Receptor Antibody

VEGF is a proangiogenic factor, and blocking its binding to its receptor inhibits proliferation, migration, survival, and angiogenesis [81]. Bevacizumab is a biologic or large molecule that acts against VEGF-A. Black box warnings issued under this drug are GI perforation and wound-healing complications. Other common side effects include bleeding, hypertension, and congestive heart failure [82].

#### 3.7.2. VEGF Inhibitors

While VEGF receptor antibodies actively bind to VEGF before it can bind to a VEGF receptor, other medications can work as decoy receptors. Aflibercept is an example of this class, and its common side effects include hypertension, dysphonia, proteinuria, hemorrhage, and increased liver function tests [83]. This medication also had a high incidence of palmer-plantar erythrodysesthesia, but this could be because it is commonly used with 5-fluorouracil, which has this side effect [84].

### 3.8. Proteasome Inhibitors

This class comprises of small molecules that function by inhibiting the chymotrypsin-like activity of the 26S proteasome in mammals [85]. Bortezomib is a small molecule proteasome inhibitor with associated adverse reactions such as myelosuppression, peripheral neuropathy, hematological toxicities, and diarrhea [86]. Peripheral neuropathy is proposed to occur through mitochondrial dysfunction that generates ROS [87].

### 3.9. Immunotherapy

Immunotherapy is one of the most interesting types of chemotherapy, which includes both large and small molecule agents.

#### 3.9.1. Immunomodulatory Small Molecules

While most immunomodulatory drugs currently in use are large molecules, there are still few older small molecule drugs such as thalidomide, lenalidomide, and pomalidomide. The most common agent, thalidomide, has a black box warning for extreme embryo–fetal toxicity [88]. Disruption in angiogenesis is by far the most important mechanism behind this fetotoxicity [89]. Another black box warning is venous thromboembolic events, and hence, patients who are at risk are advised to take this drug with anticoagulation therapy [90].

#### 3.9.2. Immune Checkpoint Inhibitors

There are several subclasses of immune checkpoint inhibitors, and they are mainly biologics or large molecules. The first subclass is the CTLA-4 inhibitor that suppresses T cells in the beginning of their activation to prevent autoimmunity [91]. Ipilimumab is a CTLA-4 inhibitor that has a black box warning for severe and fatal immune-mediated adverse reactions and can happen in any system in the body. These reactions include but are not limited to enterocolitis, hepatitis, dermatitis, and neuropathy. This follows from its mechanism of action to unleash a plethora of T cells on the body [92]. Another subclass includes PD-1 and PD-L1 inhibitors, and pembrolizumab, a popular drug in this class, can demonstrate the same variety of immune-mediated toxicities as Ipilimumab but does not have any black box warning [93].

#### 3.9.3. Cluster of Differentiation (CD)-Targeted Monoclonal Antibodies

The drugs in this class are biologics, and rituximab is the most common monoclonal antibody in this class that targets the CD20 antigen on the surface of B cells, which are overexpressed in certain cancers [94]. The black box warnings of rituximab encompass fatal infusion reactions, tumor lysis syndrome, severe mucocutaneous reactions, and multifocal leukoencephalopathy [95]. It is also worth mentioning that it can cause HBV reactivation, and prior history of hepatitis virus infection should be considered before therapy [96]. The next one is alemtuzumab that binds to CD-52 protein on lymphocytes and mediates their destruction [97]. The drug has the standard infusion black box warning as well as for pancytopenia. Although the exact mechanism of cytopenia is unknown, it may be due to the binding of bone marrow cells. Infection with herpes virus is also common with this agent [98]. The third monoclonal antibody is daratumumab, which binds to CD-38 overexpressing cells [99]. Although this agent is devoid of any black box warning, it displayed incidences of infusion reaction, neutropenia, and thrombocytopenia [100]. Elotuzumab is the last drug of this class that binds to natural killer cells through signaling lymphocytic activation molecule family member 7 (SLAMF7) and binds to CD 16 on the natural killer cell to help it find and attack the SLAMF7 that are expressed on malignant cells [101]. There is no black box warning for elotuzumab; however, the common adverse reactions are infusion reactions, opportunistic infection, and hematological toxicity [102].

#### 3.9.4. Bispecific Monoclonal Antibody

Blinatumomab is a unique monoclonal antibody that binds to CD19 on the surface of tumor cells and CD3 on the surface of T-cells; thereby, it allows the T-cell to lyse the tumor cells [103]. This is a biologic that carries black box warnings for fatal infusion reactions and neurological toxicities Other adverse effects are tumor lysis syndrome, neutropenia, and liver damage [104].

#### 3.9.5. Antibody–Drug Conjugates

Antibody–drug conjugates are also biologics that are used in both hematological and solid tumors. The first conjugate is ibritumomab tiuxetan, which binds to the same CD 20 antigen that rituximab binds to, except it has tiuxetan, which is a chelator for yttrium 90 (Y-90). Then, Y-90 emits free radicals, which damage the malignant B-cells [105]. This medication has the black box warnings for fatal infusion reactions, prolonged and severe cytopenia, and severe cutaneous and mucocutaneous reactions [106]. There are also black box warnings to not exceed 1184 MBq and to not administer to patients with altered biodistribution [107]. This is understandable considering the radioactive nature of the medication. The second conjugate is brentuximab vedotin, which by binding to CD30 allows monomethyl auristatin E (MMAE) to be transported into the tumor cells, where it acts as a microtubular disruptor that causes the cells to undergo apoptosis [108]. Its black box warning includes progressive multifocal leukoencephalopathy due to a weakened immune system being attacked by the John Cunningham virus [109]. Other adverse reactions include infusion reactions, hematological toxicities such as neutropenia, peripheral neuropathy, infusion-related reactions, and diarrhea [110].

#### 3.9.6. Cytotoxin–Protein Conjugate

Cytotoxin–protein conjugates can be useful when the target of the drug is interleukin instead of an antigen. Denileukin diftitox is indicated in the treatment of cutaneous T-cell lymphoma. It has black box warnings for fatal infusion reactions, capillary leak syndrome, and loss of visual acuity [111].

## 4. Comparison between Toxicities from the Old (Small Molecules) Drugs and New (Biologics) Drugs

**Figure 2:**
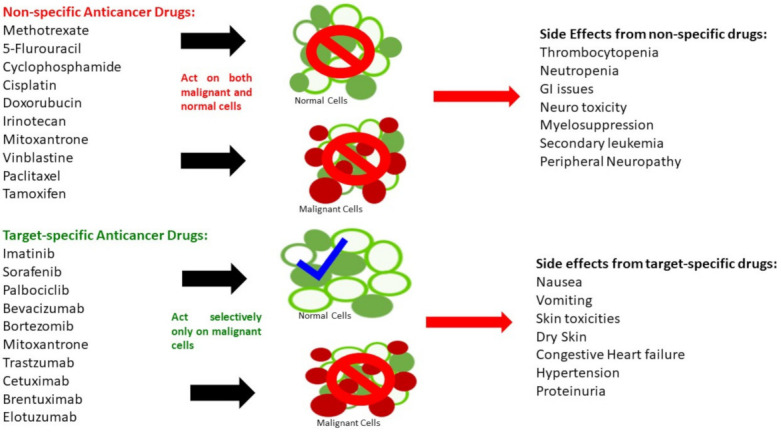
General mechanism of action and adverse effects of nonspecific vs. targeted anticancer drugs.

The oncologic drugs encompass a wide range of toxicities, and each of them has their own advantages and limitations. A pivotal distinguishing feature between small molecule drugs and biologics is based on the way they interact with their targets and produce the outcome after administration. Small molecules can interact with both malignant and normal cells and hence can produce frequent adverse effects. Basically, these drugs can obstruct basal physiological functions and initiate lethal complications. Compared to small molecule drugs, the biologics or large molecules are usually more target-specific, which leads to more specific binding/interaction with their targets on cell surfaces or intracellular components. Thus, they result in relatively less nonspecific adverse effects; however, the adverse effects can still be life threatening. Figure 2 shows the general mechanisms of action and adverse effects of both types of agents.

**Figure 3:**
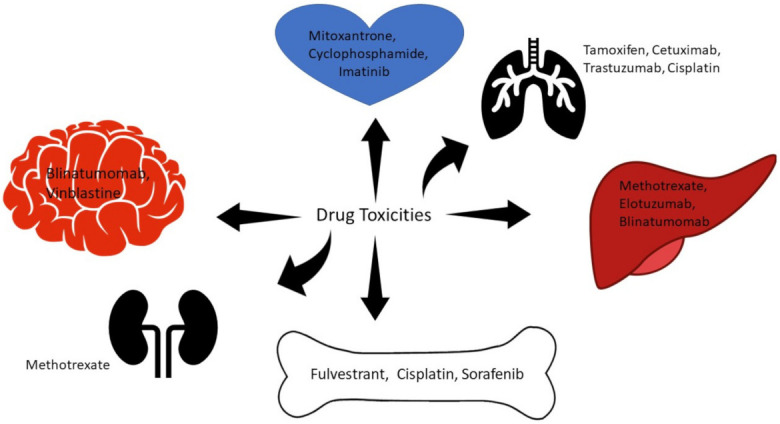
Organ-based adverse effects of anticancer drugs with representative examples.

It is important to recognize the common points between small and large molecules and to distinguish different toxicities such as dermatologic, cardiotoxicities, myelosuppression, peripheral neuropathy, pulmonary toxicity, and hepatotoxicity. Figure 3 summarizes organ-based toxicities of the commonly used anticancer agents.

### 4.1. Dermatologic Toxicities

When discussing dermatologic adverse reactions, it should be noted that this is not a major characteristic of oncology drugs in general. However, it is present among certain classes. When comparing the relevance of dermatologic adverse effects in newer versus older drugs, it is more commonly seen in the newer and large molecule drugs such as panitumumab and cetuximab [112]. However, one class of smaller molecules exhibits dermatologic reactions as well such as gefitinib, erlotinib, sorafenib, and afatinib [113]. When analyzing the degree of toxicity, the larger molecules panitumumab and cetuximab had much higher dermatologic toxicities. Panitumumab in its first clinical trial had skin toxicities in around 89% of patients, and cetuximab also showed rash in 27.85% of patients in its second clinical trial, erbitux in first-line treatment of recurrent or metastatic head and neck cancer (EXTREME) [114]. Mechanistically, an elevated expression of cytokines such as tumor necrosis factor-alpha, interleukin-1 alpha, and interferon-gamma are reported to be the contributing factors for dermal toxicity [115].

### 4.2. Cardiotoxicities

Alkylating agents and anthracenediones are known to cause cardiotoxicities [116]. When observing their indications, the only commonality between the older agents is in the treatment of leukemia [117]. For example, cyclophosphamide, a common nitrogen mustard alkylating agent, has many cardiotoxicities including myocarditis, myopericarditis, pericardial effusion, arrythmias, and congestive heart failure [118]. Interestingly, mitoxantrone has a notable cardiotoxicity, and its severity is much worse with a black box warning for congestive heart failure. Doxorubicin is also reported to cause severe cardiotoxicity. All these drugs lead to cardiotoxicity through ROS production [119]. Arsenic trioxide is another agent that produces cardiac complications. Its black box warnings include differentiation syndrome and cardiac arrhythmia that is caused by prolonged QT interval. The mechanism of this prolonged QT interval is due to an extended duration of action potential, which implies that arsenic trioxide has a direct impact on cardiac repolarization [120]. When turning to newer agents such as the BCR-ABL inhibitors, imatinib also carries precautions for congestive heart failure [121]. This drug can result in cardiotoxicity through the inhibition of mitochondrial protection pathways. The medications to treat both breast and prostate cancers carry cardiotoxicity warnings [122]. This is because hormonal regulation affects cardiovascular health with their ability to produce free radicals that can damage the myocardium. All the SERMs have a contraindication for patients who have had a venous thromboembolism [66]. Patients taking an anticoagulant should not take SERMs. Abiraterone has been shown to cause cardiac failure, grade 3 or 4 in 1.9% of patients in phase 3 trials and should be used with caution [123]. Hence, cardiotoxicity is predominantly observed in small molecules where ROS generation plays a critical role.

### 4.3. Bone Marrow Suppression, Myelosuppression

Bone marrow suppression and myelosuppression are very common in many classes, both older and newer drugs; however, they differ in severity [124]. Fulvestrant, an SERD used in the treatment of breast cancer, is reported to cause bone pain. This drug has 100 times more binding affinity than tamoxifen [125]. When comparing it to the similar class of SERMS, tamoxifen is basically an agonist in bone and is not associated with this toxicity [126]. In very old drugs such as alkylating agents, both cyclophosphamide and cisplatin are known to cause myelosuppression [43]. However, cisplatin causes a more severe toxicity and carries a black box warning [47]. In case of antitumor antibiotics, drugs in both anthracycline and camptothecin subclasses demonstrate myelosuppression [127]. However, the myelosuppression in irinotecan is more severe than that in doxorubicin. Although both are classified as antitumor antibiotics, they differ in their mechanisms of action, and this could be the reason for the range of effect on the cells. Regarding antimetabolite agents, methotrexate carries a black box warning for bone marrow suppression because of its effect on cells in proliferating tissues [128]. When shifting toward newer medications, subclasses affecting the tyrosine pathway are associated with myelosuppression. These medications are used to treat leukemia, and therefore, myelosuppression would be a predictable adverse effect [129]. These subclasses include the BCR-ABL inhibitors such as imatinib and the RAF inhibitors including sorafenib. When compared to cytarabine, an older medication used to treat leukemia, imatinib showed significantly less neutropenia and thrombocytopenia [121]. In fact, older agents carry a higher degree of severity, as they work less specifically.

### 4.4. Peripheral Neuropathy

A wide variety of oncologic drugs, both newer and older agents, are known to cause peripheral neuropathy. For example, old agents such as paclitaxel, cisplatin, and doxorubicin and newer agents such as trastuzumab and bortezomib cause peripheral neuropathy [56]. Paclitaxel causes this adverse reaction by inducing mitotic changes in the cell process [130]. A newer agent, trastuzumab has exhibited peripheral neuritis and neuropathy in over 5% of patients. However, when combined with paclitaxel, the incidence increases by over 10 times [131]. Peripheral neurotoxicity has also been reported with doxorubicin; however, it is not due to its mechanism, rather its route of administration when given intra-arterially [132]. It is important to understand that although different classes may carry similar toxicities, they all work differently and can be affected by route, mechanism, dose, and many other factors. Overall, mitochondrial dysfunction as well as mitotoxicity and oxidative stress contribute to this peripheral neuropathy [56].

### 4.5. Pulmonary Toxicities

With older agents such as tamoxifen, there is a black box warning for pulmonary embolism because of its ability to mimic estrogen in certain tissues, causing an increased risk of blood clots [133]. Bleomycin and methotrexate produce interstitial infiltrates and cyclophosphamide generates alveolar damage [134]. In newer drugs, the most known class for causing pulmonary toxicities includes the EGFR antagonists. Cetuximab is reported to cause cardiopulmonary arrest, particularly when given with cisplatin, which is a much older agent [135]. This reaction is caused by an electrolyte imbalance. Trastuzumab also carries the risk of pulmonary toxicities resulting from infusion [16,72,131]. All these agents cause either alveolar damage, pulmonary hemorrhage, or hypersensitivity pneumonitis [134].

### 4.6. Hepatotoxicity

Hepatotoxicity is a very important adverse reaction because the liver is primarily responsible for the metabolism and drug interactions. Without a functionable liver, a patient’s treatment options get decreased, and as such, it is imperative to monitor the drugs causing hepatotoxicity [136]. Methotrexate, the most common antimetabolite, has been reported to cause hepatotoxicity by activating the inflammatory pathways and cytokines and ROS production. Doxorubicin-induced membrane lipid peroxidation and disrupted mitochondrial energy metabolism can also result in liver damage [41]. In contrast to this old generation drug, newer monoclonal antibodies also have warnings for hepatotoxicity. For example, in clinical studies involving elotuzumab, there were elevated LFT’s within three times the upper normal limits in 7.6% of patients. However, unlike the fatal relationship shown in methotrexate, six out of eight patients on elotuzumab were able to resolve the liver toxicity and continue with treatment, and only one patient had LFTs over three times the upper normal limit [82]. Falling under the same general class, the bispecific monoclonal antibody blinatumomab is also associated with liver toxicities. Elevation in liver enzymes occurred in around 6% of patients, and only 1% of patients had to discontinue treatment because of this reaction [137]. Another class that is known for causing liver problems includes BCR-ABL inhibitors. Imatinib showed fatal hepatotoxicity as well as severe reactions [138]. Broadly, ROS production, lipid peroxidation, expression of pro-inflammatory genes, and immune cell hyperactivation are linked to drug-induced hepatotoxicity.

### 4.7. Nephrotoxicity

Nephrotoxicity is another vital adverse reaction, since a significant number of patients receiving chemotherapy demonstrate compromised renal function. Furthermore, many of these patients already have underlying renal abnormalities that may worsen and complicate treatment outcome. Hence, it is critical to make dose adjustments and carefully monitor the renally compromised patients. Small molecules such as methotrexate results in acute renal failure due to the precipitation of the agent as well as 7-hydroxymethotrexate into the renal tubules. The concomitant use of methotrexate with other nephrotoxic agents such as cisplatin may disrupt their excretion, leading to the development of severe myelosuppression [38]. Cisplatin itself is a strong nephrotoxic that elicits damage to the loop of Henle, s3 segment of proximal tubule, and distal tubules. The activation of MAP kinase, p53, and DNase 1 pathways along with an increase in tumor necrosis factor α (TNFα) facilitates cisplatin-induced nephrotoxicity [39]. Cyclophosphamide produces hemorrhagic cystitis that is closely linked to hyponatremia. Biologics such as bevacizumab and cetuximab were also reported to cause nephrotoxicity. Bevacizumab can produce proteinuria through immune complex-mediated focal glomerulonephritis, and cetuximab can result in hypomagnesemia by deactivating the TRPM6 magnesium channel. Essentially, both small molecules and biologics affect the vasculature and glomerular perfusion of the nephron that impairs renal excretion and thereby results in nephrotoxicity [139].

## 5. CYP/Transporter in Chemotherapy Toxicities

There is a significant difference between the way the small and large molecules are processed by patients. Transporter proteins are involved in moving the small molecule anticancer drugs across the membrane, but large molecule antibodies are independent of transports as they are intravenously administered. Since transporters are strategically located in the key locations of major ADME organs intestine, renal, and hepatic epithelial, and in other anticancer drug targets such as brain and lung, these protein influence both the pharmacokinetics and pharmacodynamics of small molecular anticancer drugs [140]. The efflux (e.g., P-gp) and uptake (e.g., OCT) transporters have an opposing role in influencing toxicity from anticancer drugs [141]. Co-administered drugs with anticancer drugs such as azole antifungals, ritonavir, and cyclosporine can inhibitor P-gp and increase plasma concentration [142]. Several anticancer drugs are substrate of efflux transports; examples include vincristine, doxorubicin, and taxanes [143]. These transporters can cause resistance due to their ability to remove the drug from the cancerous cells or tumor. Inducers of P-gp such as rifampin and St. John’s wort have the ability to increase the efflux capability of the anticancer drugs, leading to suboptimal drug levels in the tumors or cancer cells [142]. In general, even without the presence of any inducers, P-gp protein levels are overexpressed in cancerous conditions, especially in the resistance or metastasis stage [143]. However, inhibition of the efflux transporters opens up the opportunity to increase plasma concentration above minimum toxic concentrations.

Similarly, the metabolism of small molecules involves both phase 1 and phase 2 metabolism processes, whereas large molecules are usually metabolized into smaller peptides and amino acids by non-CYP processes [144]. CYP enzymes are a superfamily of enzymes involved in the phase I metabolism of small molecule anticancer drugs [145]. CYP inhibitors and inducers can cause substantial changes in the concentrations of these small molecules, which in turn can raise a therapeutic dose into the toxic range or push a therapeutic dose into a subtherapeutic range. A common antifungal class used for prophylaxis in many chemotherapeutic regimens is azole antifungals. These antifungal agents are potent CYP inhibitors that inhibit CYP2C9 and CYP3A4 [146]. As shown in Table 3, many of the small molecules are metabolized by CYP3A4. Thus, many of these medications can reach toxic doses when co-administered with an azole antifungal [147]. On the contrary, CYP inducers can accelerate the excretion of small molecules and thus cause sub-therapeutic plasma and/or cellular concentration of anticancer drugs [148]. In several instances, the anticancer drug dose needs to be recalculated if the patient is taking an azole antifungal; however, the amount of inhibition can vary from person to person depending on the diet, age, and health status.

## 6. Role of Pharmacogenomics in Differential Toxic Effects

Genetic differences in ADME genes can significantly contribute toward the interindividual differences in toxic effects of anticancer drugs. Since ADME proteins (e.g., transporters, CYPs, carbonyl reductases, UGTs) are more relevant to the disposition of small molecule anticancer drugs, the genetic changes have pronounced effects on the transport and metabolism of small molecule anticancer drugs [152]. Germline variants are responsible for ADME-related pharmacogenomics of anticancer drugs. Since oncologic drugs typically have narrow therapeutic windows, a slight genetic variation between patients can mean life or death for these patients. For small molecules, most of the emphasis is placed on CYP and UGT enzymes, transferases, and transport proteins [153]. CYP enzymes and transferases metabolize medications and prepare them for excretion. Different CYP enzymes that break down medications were highlighted in the last section. There are many genetic polymorphisms that cause CYP enzymes to work either more effectively or in most cases less effectively. The most common CYP enzyme that is involved in small molecule metabolism is CYP3A4 [149]. It has been previously observed that genetic polymorphisms such as the single nucleotide polymorphism CYP3A4*22 account for up to 88% of individual variation of CYP3A4 metabolism [154]. If patients with one of these polymorphisms on the CYP3A4 gene have a medication that is a substrate of CYP3A4, they will not efficiently metabolize that medication. Drugs such as doxorubicin can be lethal if the genetic polymorphism in carbonyl reductase is not detected. CYP3A4*1B occurs in 2–9% of white males and in even higher numbers in African Americans. The *4 variation in the CYP2D6 allele also causes changes in breast cancer treatment through lowering tamoxifen efficacy [155]. 5-Flurouracil is another drug that is highly susceptible by genetic variations. Individuals with the *2 polymorphism on their dihydropyrimidine dehydrogenase gene can experience elevated toxicity due to their inability to break down 5-Flurouracil [156].

Transporter proteins allow the medication to get in and out of tissues. In cancer, P-gp is the primary transporter that has caused several therapeutic challenges. This protein is also known as the Multi-Drug Resistance Protein or MRP1. It is because it pumps the drug out of the tumor cells before the drug can work [157]. These proteins can be overexpressed during oncological drug therapy and can cause tolerance and resistance to the drug therapy. If patients are already predisposed because of their genetics to having a higher level of P-gp, then it can cause a treatment to become ineffective. There are also influx transporters that are involved in drug toxicities [158]. The organic cation transporter 2 (OCT2) causes nephrotoxicity in patients that are treated with cisplatin. If this transporter is overexpressed due to genetic factors, then it can cause excess toxicity. Wilson et al. (2017) established several polymorphisms causing a change of expression for South African Tribes [159]. Some polymorphisms led to small changes in OCT2 expression, but some led to large changes. These changes, both increasing and decreasing the OCT2 expression, could be significant for patients receiving cisplatin therapy and their resulting nephrotoxicity [46]. For large molecules, there is a lot of new research determining which genetic predispositions can affect patient treatment.

## 7. Summary

In recent times, the pharmaceutical and biomedical research community is focusing more on the biologics considering the greater specificity of the molecules. The primary targets of the biologics are secreted or cell surface proteins. Therefore, proteomics could be helpful to identify novel targets for these molecules and concomitantly demonstrate fewer adverse effects [160]. Lately, ligandomics has emerged as another unique strategy that can discover malignancy-related novel ligands as therapeutic targets and ultimately generate less toxic biologics [161]. The genomics techniques are already contributing to the sequencing of the genome of interest to characterize the mutational landscapes of cancer patients. Moreover, metabolomics is another fresh avenue that can detect and quantify novel oncometabolites that may serve as important biomarkers in different types of cancers [162]. All these OMICS studies may pave a way to better understand the pathophysiology of cancer and develop novel anticancer chemotherapeutics that are devoid of major life-threatening toxicities.

This review is intended to compare the toxicities between large and small anticancer drug molecules. A limitation of this review is that cost was not a factor of analysis in determining whether the benefits outweigh the risks of toxicity associated with the medications. From a toxicity standpoint, it seems that the newer agents produce less severe toxicities and therefore should be given priority over the old agents. Another area of difficulty is that newer medications have less post market trials than older agents, and less long-term effects are known due to their recent arrival to the market. All in all, the small molecules have a greater variability due to their pharmacokinetic properties. These medications, because of their narrow therapeutic window, are sensitive to minor fluctuations in their concentrations.

A complete understanding of toxicity/adverse effects of anticancer drugs, whether old or new, is crucial to design a more effective drug combination. This is also relevant to construe the toxicological profile of new chemical entities. Usually, these drugs are assessed at the maximum tolerated dose levels where the adverse effects of these compounds are a frequent manifestation of their mechanisms of action as well as their impact on growing non-cancerous cells such as stem cells, hair follicle cells, GI epithelial cells, etc. So far, the evidence suggests that large molecules may not be as unpredictable in their active drug concentration levels and therefore could be much safer in therapy. Even with all the excitement about large molecules and new mechanisms of action, drugs are still being developed with old mechanisms of action. There are pros and cons to every large paradigm shift in medication from vaccination to the first cancer treatments; however, this could be a golden age for reducing adverse effects in cancer patients due to these large molecules.

## Figures and Tables

**Table 1:** Timeline of key cancer treatment agents. Compiled from: [13,14,15].

Year	Advancement
1903	Radiation first used to cure patients of basal carcinoma of the skin
1941	Charles Huggins used castration and estrogen therapy to treat prostate cancer
1947	Anti-metabolites were first used
1949	Alkylating agents were first used
1958	First combination therapy was used (6-mercaptopurine and methotrexate)
1978	Tamoxifen approved for birth control
1996	First aromatase inhibitor is approved
1997	First large molecule for cancer treatment rituximab was approved
1998	Trastuzumab was approved
2001	Imatinib is approved
2011	Ipilimumab is approved
2014	Pembrolizumab is approved

**Table 2:** Mechanistic class, drug, and prototype adverse effect(s) of representative chemotherapeutic agents. Compiled from: [16,17,18,19,20,21,22,23,24,25,26,27,28,29,30,31,32,33,34,35,36,37].

Class	Mechanistic Class	Drug	Main Adverse Effect
Alkylating Agents	Nitrogen Mustards	Cyclophosphamide	Myelosuppression
Antimetabolites	Pyrimidine Analogs	Fluorouracil	Leukopenia
		Capecitabine	Diarrhea
	Antifolates	Methotrexate	Renal Failure (especially with cisplatin)
	Other Antibiotics	Mitomycin	Leukopenia
Topoisomerase Inhibitor	Camptothecins	Irinotecan	Diarrhea
Antitumor Antibiotics	Anthracyclines	Doxorubicin	Cardiac Toxicity
		Dactinomycin	Cardiac Toxicity
Antimitotic Agents	Taxanes/Epothilones	Paclitaxel	Neutropenia
	Vinca Alkaloids	Vincristine	Leukopenia
Hormonal Agents	SERMs	Tamoxifen	Embolism
		Raloxifene	Embolism
	Antiestrogens	Fulvestrant	Hot Flashes
	Aromatase Inhibitors	Anastrozole	Hot Flashes
	Antiandrogens	Enzalutamide	Fatigue
		Abiraterone	Adrenocortical Insufficiency
		Apalutamide	Fatigue
	GnRH Antagonists	Abarelix	QT Prolongation
		Degarelix	QT Prolongation
	Organoplatinum Complexes	Cisplatin	Renal Failure
EGFR Antagonists	EGFR (ErbB1) Blockers	Cetuximab	Cardiopulmonary Arrest
	HER2 (ErbB2) Blockers	Trastuzumab, Ado-trastuzumab Emtansine (Antibody Drug Conjugate)	Cardiomyopathy
	Tyrosine Kinase Inhibitors	Sunitinib	Diarrhea
		Afatinib	Diarrhea
		Gefitinib	Diarrhea
Kinase and Signal Transduction Antagonists	CDK 4 and 6 Inhibitor	Palbociclib	Neutropenia
	Anaplastic Lymphoma Kinase (ALK) Inhibitor	Ceritinib	GI Toxicity
	Janus-Associated Kinases (JAKs) inhibitor	Ruxolitinib	Thrombocytopenia
	Phosphatidylinositol 3-kinase Inhibitor	Idelalisib	Hepatic Toxicity
	Immune Checkpoint (CTLA-4/PD-1) Inhibitors	Pembrolizumab	Pneumonitis

**Table 3:** Representative small molecule chemotherapeutic agents as the substrates of cytochrome P450 enzymes and transporters. Compiled from: [142,145,146,149,150,151].

Oncology Drug	CYP Substrate	Induction	Inhibition	Transporter
Cyclophosphamide	CYP2B6, CYP3A4, CYP2D6	CYP3A4	N/A	
Cytarabine	CYP3A4		CYP3A4	
Doxorubicin	CYP2D6, CYP3A4	N/A	CYP2D6	P-gp, OCT6
Irinotecan	CYP3A4	CYP3A4		P-gp, OCT3
Vincristine	CYP3A4		CYP2D6	P-gp, OATP1B1
Vinblastine	CYP3A4		CYP2D6	P-gp, OATP1B1
Paclitaxel	CYP2C8, CYP3A4	CYP3A4		P-gp, OATP1B1
Docetaxel	CYP3A4			P-gp, OATP1B3
Enzalutamide	CYP2C8, CYP3A4	CYP2C19, CYP2C9,CYP3A4		
Abiraterone	CYP3A4		CYP2C8, CYP2D6, CYP3A4	
Tamoxifen	CYP2D6, CYP3A4, CYP2C9		CYP3A4	
Anastrozole	CYP3A		CYP1A2, CYP2C8, CYP2C9, CYP3A4	
Letrozole	CYP2A6, CYP3A4		CYP2A6, CYP2C19	
Gefitinib	CYP3A4		CYP2C19, CYP2D6	P-gp, BCRP, OATP1B3
Erlotinib	CYP3A4, CYP1A2			P-gp, BCRP, OAT3
Imatinib mesylate	CYP3A4		CYP2C9, CYP2D6, CYP3A4	P-gp, BCRP, OCT1
Gefitinib	CYP3A4		CYP2C19, CYP2D6	
Nilotinib	CYP3A4		CYP2C8, CYP2C9, CYP2D6	
Pazopanib	CYP3A4		CYP3A4, CYP2D6	OATP1B1
Vemurafenib	CYP3A4		CYP1A2	

## Data Availability

Data sharing not applicable.

## References

[1] World Health Organization (WHO) Cancer. https://www.who.int/news-room/fact-sheets/detail/cancer.

[2] Lee Y.T., Tan Y.J., Oon C.E. (2018). Molecular targeted therapy: Treating cancer with specificity. Eur. J. Pharmacol..

[3] Meegan M.J., O’Boyle N.M. (2019). Special Issue “Anticancer Drugs”. Pharmaceuticals.

[4] Kundranda M.N., Niu J. (2015). Albumin-bound paclitaxel in solid tumors: Clinical development and future directions. Drug Des. Dev. Ther..

[5] Tsimberidou A.M., Fountzilas E., Nikanjam M., Kurzrock R. (2020). Review of precision cancer medicine: Evolution of the treatment paradigm. Cancer Treat. Rev..

[6] Huggins C., Hodges C.V. (2002). Studies on prostatic cancer: I. The effect of castration, of estrogen and of androgen injection on serum phosphatases in metastatic carcinoma of the prostate. J. Urol..

[7] Simone J.V. (2006). History of the treatment of childhood ALL: A paradigm for cancer cure. Best Pract. Res. Clin. Haematol..

[8] Chabner B.A., Roberts T.G. (2005). Timeline: Chemotherapy and the war on cancer. Nat. Rev. Cancer.

[9] Devita V.T., Serpick A.A., Carbone P.P. (1970). Combination chemotherapy in the treatment of advanced Hodgkin’s disease. Ann. Intern. Med..

[10] Maloney D.G., Grillo-Lopez A.J., White C.A., Bodkin D., Schilder R.J., Neidhart J.A., Janakiraman N., Foon K.A., Liles T.M., Dallaire B.K. (1997). IDEC-C2B8 (Rituximab) anti-CD20 monoclonal antibody therapy in patients with relapsed low-grade non-Hodgkin’s lymphoma. Blood.

[11] Dendukuri N., Khetani K., McIsaac M., Brophy J. (2007). Testing for HER2-positive breast cancer: A systematic review and cost-effectiveness analysis. Can. Med. Assoc. J..

[12] Alexander W. (2016). The Checkpoint Immunotherapy Revolution: What Started as a Trickle Has Become a Flood, Despite Some Daunting Adverse Effects; New Drugs, Indications, and Combinations Continue to Emerge. Pharm. Ther..

[13] U.S. Food and Drug Administration Drugs@FDA: FDA-Approved Drugs. https://www.accessdata.fda.gov/scripts/cder/daf/.

[14] CenterWatch FDA Approved Drugs. https://www.centerwatch.com/directories/1067-fda-approved-drugs.

[15] American Cancer Society Cancer Information and Resources. https://www.cancer.org/.

[16] Nemeth B.T., Varga Z.V., Wu W.J., Pacher P. (2017). Trastuzumab cardiotoxicity: From clinical trials to experimental studies. Br. J. Pharmacol..

[17] Ajayi S., Becker H., Reinhardt H., Engelhardt M., Zeiser R., von Bubnoff N., Wasch R. (2018). Ruxolitinib. Recent Results Cancer Res..

[18] Cavadias I., Rouzier R., Lerebours F., Hequet D. (2020). Hot flushes and breast cancer with positive hormone receptors: Mechanisms and management. Bull. Cancer.

[19] Crombag M.B.S., Koolen S.L.W., Wijngaard S., Joerger M., Dorlo T.P.C., van Erp N.P., Mathijssen R.H.J., Beijnen J.H., Huitema A.D.R. (2019). Does Older Age Lead to Higher Risk for Neutropenia in Patients Treated with Paclitaxel?. Pharm. Res..

[20] Debruyne F., Bhat G., Garnick M.B. (2006). Abarelix for injectable suspension: First-in-class gonadotropin-releasing hormone antagonist for prostate cancer. Future Oncol..

[21] DeCensi A., Puntoni M., Guerrieri-Gonzaga A., Caviglia S., Avino F., Cortesi L., Taverniti C., Pacquola M.G., Falcini F., Gulisano M. (2019). Randomized Placebo Controlled Trial of Low-Dose Tamoxifen to Prevent Local and Contralateral Recurrence in Breast Intraepithelial Neoplasia. J. Clin. Oncol..

[22] Finn R.S., Martin M., Rugo H.S., Jones S., Im S.A., Gelmon K., Harbeck N., Lipatov O.N., Walshe J.M., Moulder S. (2016). Palbociclib and Letrozole in Advanced Breast Cancer. N. Engl. J. Med..

[23] Hakeam H.A., Arab A., Azzam A., Alyahya Z., Eldali A.M., Amin T. (2018). Incidence of leukopenia and thrombocytopenia with cisplatin plus mitomycin-c versus melphalan in patients undergoing cytoreductive surgery (CRS) and hyperthermic intraperitoneal chemotherapy (HIPEC). Cancer Chemother. Pharmacol..

[24] Healy J.B., Moriarty M.J. (1969). Vinblastine plus vincristine. Lancet.

[25] Howard S.C., McCormick J., Pui C.H., Buddington R.K., Harvey R.D. (2016). Preventing and Managing Toxicities of High-Dose Methotrexate. Oncologist.

[26] Kassem L., Shohdy K.S., Lasheen S., Abdel-Rahman O., Ali A., Abdel-Malek R.R. (2019). Safety issues with the ALK inhibitors in the treatment of NSCLC: A systematic review. Crit. Rev. Oncol. Hematol..

[27] Khanna C., Lund E.M., Redic K.A., Hayden D.W., Bell F.W., Goulland E.L., Klausner J.S. (1998). Randomized controlled trial of doxorubicin versus dactinomycin in a multiagent protocol for treatment of dogs with malignant lymphoma. J. Am. Vet. Med. Assoc..

[28] Kittai A.S., Blank J., Graff J.N. (2018). Gonadotropin-Releasing Hormone Antagonists in Prostate Cancer. Oncology.

[29] Kwok G., Yau T.C., Chiu J.W., Tse E., Kwong Y.L. (2016). Pembrolizumab (Keytruda). Hum. Vaccin. Immunother..

[30] Manohar S., Leung N. (2018). Cisplatin nephrotoxicity: A review of the literature. J. Nephrol..

[31] Mego M., Chovanec J., Vochyanova-Andrezalova I., Konkolovsky P., Mikulova M., Reckova M., Miskovska V., Bystricky B., Beniak J., Medvecova L. (2015). Prevention of irinotecan induced diarrhea by probiotics: A randomized double blind, placebo controlled pilot study. Complement. Ther. Med..

[32] Primrose J., Falk S., Finch-Jones M., Valle J., O’Reilly D., Siriwardena A., Hornbuckle J., Peterson M., Rees M., Iveson T. (2014). Systemic chemotherapy with or without cetuximab in patients with resectable colorectal liver metastasis: The New EPOC randomised controlled trial. Lancet Oncol..

[33] Primrose J.N., Fox R.P., Palmer D.H., Malik H.Z., Prasad R., Mirza D., Anthony A., Corrie P., Falk S., Finch-Jones M. (2019). Capecitabine compared with observation in resected biliary tract cancer (BILCAP): A randomised, controlled, multicentre, phase 3 study. Lancet Oncol..

[34] Ramanathan S., Jin F., Sharma S., Kearney B.P. (2016). Clinical Pharmacokinetic and Pharmacodynamic Profile of Idelalisib. Clin. Pharmacokinet..

[35] Rugo H.S., Di Palma J.A., Tripathy D., Bryce R., Moran S., Olek E., Bosserman L. (2019). The characterization, management, and future considerations for ErbB-family TKI-associated diarrhea. Breast Cancer Res. Treat..

[36] Shajahan J., Pillai P.S., Jayakumar K.N. (2015). A Prospective Comparative Study of the Toxicity Profile of 5-Flurouracil, Adriamycin, Cyclophosphamide Regime VS Adriamycin, Paclitaxel Regime in Patients with Locally Advanced Breast Carcinoma. J. Clin. Diagn. Res..

[37] Wang Y., Chen J., Wu Z., Ding W., Gao S., Gao Y., Xu C. (2021). Mechanisms of enzalutamide resistance in castration-resistant prostate cancer and therapeutic strategies to overcome it. Br. J. Pharmacol..

[38] Widemann B.C., Adamson P.C. (2006). Understanding and managing methotrexate nephrotoxicity. Oncologist.

[39] Townsend D.M., Deng M., Zhang L., Lapus M.G., Hanigan M.H. (2003). Metabolism of Cisplatin to a nephrotoxin in proximal tubule cells. J. Am. Soc. Nephrol..

[40] Mahoney D.H., Shuster J.J., Nitschke R., Lauer S.J., Steuber C.P., Winick N., Camitta B. (1998). Acute neurotoxicity in children with B-precursor acute lymphoid leukemia: An association with intermediate-dose intravenous methotrexate and intrathecal triple therapy--a Pediatric Oncology Group study. J. Clin. Oncol..

[41] Mudd T.W., Guddati A.K. (2021). Management of hepatotoxicity of chemotherapy and targeted agents. Am. J. Cancer Res..

[42] Thomas A., Teicher B.A., Hassan R. (2016). Antibody-drug conjugates for cancer therapy. Lancet Oncol..

[43] Que L., He L., Yu C., Yin W., Ma L., Cao B., Yu S. (2016). Activation of Nrf2-ARE signaling mitigates cyclophosphamide-induced myelosuppression. Toxicol. Lett..

[44] Kenney L.B., Laufer M.R., Grant F.D., Grier H., Diller L. (2001). High risk of infertility and long term gonadal damage in males treated with high dose cyclophosphamide for sarcoma during childhood. Cancer.

[45] Ciarimboli G., Deuster D., Knief A., Sperling M., Holtkamp M., Edemir B., Pavenstadt H., Lanvers-Kaminsky C., am Zehnhoff-Dinnesen A., Schinkel A.H. (2010). Organic cation transporter 2 mediates cisplatin-induced oto- and nephrotoxicity and is a target for protective interventions. Am. J. Pathol..

[46] Ciarimboli G. (2012). Membrane transporters as mediators of Cisplatin effects and side effects. Scientifica.

[47] Quintanilha J.C.F., Saavedra K.F., Visacri M.B., Moriel P., Salazar L.A. (2019). Role of epigenetic mechanisms in cisplatin-induced toxicity. Crit. Rev. Oncol. Hematol..

[48] Fotopoulou C. (2014). Limitations to the use of carboplatin-based therapy in advanced ovarian cancer. Eur. J. Cancer Suppl..

[49] Branca J.J.V., Carrino D., Gulisano M., Ghelardini C., Di Cesare Mannelli L., Pacini A. (2021). Oxaliplatin-Induced Neuropathy: Genetic and Epigenetic Profile to Better Understand How to Ameliorate This Side Effect. Front. Mol. Biosci..

[50] Xu M.F., Tang P.L., Qian Z.M., Ashraf M. (2001). Effects by doxorubicin on the myocardium are mediated by oxygen free radicals. Life Sci..

[51] Smith R.E., Bryant J., DeCillis A., Anderson S., National Surgical Adjuvant B., Bowel Project E. (2003). Acute myeloid leukemia and myelodysplastic syndrome after doxorubicin-cyclophosphamide adjuvant therapy for operable breast cancer: The National Surgical Adjuvant Breast and Bowel Project Experience. J. Clin. Oncol..

[52] Jordan K., Behlendorf T., Mueller F., Schmoll H.J. (2009). Anthracycline extravasation injuries: Management with dexrazoxane. Ther. Clin. Risk Manag..

[53] Moukharskaya J., Verschraegen C. (2012). Topoisomerase 1 inhibitors and cancer therapy. Hematol. Oncol. Clin. N. Am..

[54] Takasuna K., Hagiwara T., Hirohashi M., Kato M., Nomura M., Nagai E., Yokoi T., Kamataki T. (1998). Inhibition of intestinal microflora beta-glucuronidase modifies the distribution of the active metabolite of the antitumor agent, irinotecan hydrochloride (CPT-11) in rats. Cancer Chemother. Pharmacol..

[55] Vile G.F., Winterbourn C.C. (1989). Microsomal lipid peroxidation induced by adriamycin, epirubicin, daunorubicin and mitoxantrone: A comparative study. Cancer Chemother. Pharmacol..

[56] Han Y., Smith M.T. (2013). Pathobiology of cancer chemotherapy-induced peripheral neuropathy (CIPN). Front. Pharmacol..

[57] Martino E., Casamassima G., Castiglione S., Cellupica E., Pantalone S., Papagni F., Rui M., Siciliano A.M., Collina S. (2018). Vinca alkaloids and analogues as anti-cancer agents: Looking back, peering ahead. Bioorg. Med. Chem. Lett..

[58] Florian S., Mitchison T.J. (2016). Anti-Microtubule Drugs. Anti-Microtubule Drugs.

[59] Ho M.Y., Mackey J.R. (2014). Presentation and management of docetaxel-related adverse effects in patients with breast cancer. Cancer Manag. Res..

[60] Nightingale G., Ryu J. (2012). Cabazitaxel (jevtana): A novel agent for metastatic castration-resistant prostate cancer. Pharm. Ther..

[61] Castells M. (2017). Drug Hypersensitivity and Anaphylaxis in Cancer and Chronic Inflammatory Diseases: The Role of Desensitizations. Front. Immunol..

[62] Solomon Z.J., Mirabal J.R., Mazur D.J., Kohn T.P., Lipshultz L.I., Pastuszak A.W. (2019). Selective Androgen Receptor Modulators: Current Knowledge and Clinical Applications. Sex. Med. Rev..

[63] Sanford M. (2013). Enzalutamide: A review of its use in metastatic, castration-resistant prostate cancer. Drugs.

[64] De Bono J.S., Logothetis C.J., Molina A., Fizazi K., North S., Chu L., Chi K.N., Jones R.J., Goodman O.B., Saad F. (2011). Abiraterone and increased survival in metastatic prostate cancer. N. Engl. J. Med..

[65] Zhu X., Wu S. (2019). Risk of hypertension in Cancer patients treated with Abiraterone: A meta-analysis. Clin. Hypertens..

[66] Patel H.K., Bihani T. (2018). Selective estrogen receptor modulators (SERMs) and selective estrogen receptor degraders (SERDs) in cancer treatment. Pharmacol. Ther..

[67] Khan Q.J., O’Dea A.P., Sharma P. (2010). Musculoskeletal adverse events associated with adjuvant aromatase inhibitors. J. Oncol..

[68] Hamadeh I.S., Patel J.N., Rusin S., Tan A.R. (2018). Personalizing aromatase inhibitor therapy in patients with breast cancer. Cancer Treat. Rev..

[69] Pozzi C., Cuomo A., Spadoni I., Magni E., Silvola A., Conte A., Sigismund S., Ravenda P.S., Bonaldi T., Zampino M.G. (2016). The EGFR-specific antibody cetuximab combined with chemotherapy triggers immunogenic cell death. Nat. Med..

[70] Yang J.C., Sequist L.V., Geater S.L., Tsai C.M., Mok T.S., Schuler M., Yamamoto N., Yu C.J., Ou S.H., Zhou C. (2015). Clinical activity of afatinib in patients with advanced non-small-cell lung cancer harbouring uncommon EGFR mutations: A combined post-hoc analysis of LUX-Lung 2, LUX-Lung 3, and LUX-Lung 6. Lancet Oncol..

[71] Slamon D.J., Godolphin W., Jones L.A., Holt J.A., Wong S.G., Keith D.E., Levin W.J., Stuart S.G., Udove J., Ullrich A. (1989). Studies of the HER-2/neu proto-oncogene in human breast and ovarian cancer. Science.

[72] Mohan N., Jiang J., Dokmanovic M., Wu W.J. (2018). Trastuzumab-mediated cardiotoxicity: Current understanding, challenges, and frontiers. Antib. Ther..

[73] Egloff H., Kidwell K.M., Schott A. (2018). Ado-Trastuzumab Emtansine-Induced Pulmonary Toxicity: A Single-Institution Retrospective Review. Case Rep. Oncol..

[74] Cersosimo R.J. (2006). Gefitinib: An adverse effects profile. Expert Opin. Drug Saf..

[75] Burmeister T., Schwartz S., Bartram C.R., Gokbuget N., Hoelzer D., Thiel E., GMALL Study Group (2008). Patients’ age and BCR-ABL frequency in adult B-precursor ALL: A retrospective analysis from the GMALL study group. Blood.

[76] Paech F., Bouitbir J., Krahenbuhl S. (2017). Hepatocellular Toxicity Associated with Tyrosine Kinase Inhibitors: Mitochondrial Damage and Inhibition of Glycolysis. Front. Pharmacol..

[77] Jabbour E., Deininger M., Hochhaus A. (2011). Management of adverse events associated with tyrosine kinase inhibitors in the treatment of chronic myeloid leukemia. Leukemia.

[78] Li Y., Gao Z.H., Qu X.J. (2015). The adverse effects of sorafenib in patients with advanced cancers. Basic Clin. Pharmacol. Toxicol..

[79] Thill M., Schmidt M. (2018). Management of adverse events during cyclin-dependent kinase 4/6 (CDK4/6) inhibitor-based treatment in breast cancer. Ther. Adv. Med. Oncol..

[80] Gong J., Cho M., Yu K.W., Waisman J., Yuan Y., Mortimer J. (2018). A single institution experience with palbociclib toxicity requiring dose modifications. Breast Cancer Res. Treat..

[81] Niu G., Chen X. (2010). Vascular endothelial growth factor as an anti-angiogenic target for cancer therapy. Curr. Drug Targets.

[82] Garcia J., Hurwitz H.I., Sandler A.B., Miles D., Coleman R.L., Deurloo R., Chinot O.L. (2020). Bevacizumab (Avastin(R)) in cancer treatment: A review of 15 years of clinical experience and future outlook. Cancer Treat. Rev..

[83] Gaya A., Tse V. (2012). A preclinical and clinical review of aflibercept for the management of cancer. Cancer Treat. Rev..

[84] Tang P.A., Moore M.J. (2013). Aflibercept in the treatment of patients with metastatic colorectal cancer: Latest findings and interpretations. Ther. Adv. Gastroenterol..

[85] Orlowski M., Wilk S. (2000). Catalytic activities of the 20 S proteasome, a multicatalytic proteinase complex. Arch. Biochem. Biophys..

[86] Richardson P.G., Anderson K.C. (2003). Bortezomib: A novel therapy approved for multiple myeloma. Clin. Adv. Hematol. Oncol..

[87] Landowski T.H., Megli C.J., Nullmeyer K.D., Lynch R.M., Dorr R.T. (2005). Mitochondrial-mediated disregulation of Ca^2+^ is a critical determinant of Velcade (PS-341/bortezomib) cytotoxicity in myeloma cell lines. Cancer Res..

[88] Lenz W., Knapp K. (1962). Thalidomide embryopathy. Arch. Environ. Health.

[89] Therapontos C., Erskine L., Gardner E.R., Figg W.D., Vargesson N. (2009). Thalidomide induces limb defects by preventing angiogenic outgrowth during early limb formation. Proc. Natl. Acad. Sci. USA.

[90] Scarpace S.L., Hahn T., Roy H., Brown K., Paplham P., Chanan-Khan A., van Besien K., McCarthy P.L. (2005). Arterial thrombosis in four patients treated with thalidomide. Leuk. Lymphoma.

[91] Parry R.V., Chemnitz J.M., Frauwirth K.A., Lanfranco A.R., Braunstein I., Kobayashi S.V., Linsley P.S., Thompson C.B., Riley J.L. (2005). CTLA-4 and PD-1 receptors inhibit T-cell activation by distinct mechanisms. Mol. Cell. Biol..

[92] Almutairi A.R., McBride A., Slack M., Erstad B.L., Abraham I. (2020). Potential Immune-Related Adverse Events Associated With Monotherapy and Combination Therapy of Ipilimumab, Nivolumab, and Pembrolizumab for Advanced Melanoma: A Systematic Review and Meta-Analysis. Front. Oncol..

[93] Martins F., Sofiya L., Sykiotis G.P., Lamine F., Maillard M., Fraga M., Shabafrouz K., Ribi C., Cairoli A., Guex-Crosier Y. (2019). Adverse effects of immune-checkpoint inhibitors: Epidemiology, management and surveillance. Nat. Rev. Clin. Oncol..

[94] Cerny T., Borisch B., Introna M., Johnson P., Rose A.L. (2002). Mechanism of action of rituximab. Anticancer Drugs.

[95] Looney R.J. (2009). Update on the use of rituximab for intractable rheumatoid arthritis. Open Access Rheumatol..

[96] Kusumoto S., Arcaini L., Hong X., Jin J., Kim W.S., Kwong Y.L., Peters M.G., Tanaka Y., Zelenetz A.D., Kuriki H. (2019). Risk of HBV reactivation in patients with B-cell lymphomas receiving obinutuzumab or rituximab immunochemotherapy. Blood.

[97] Jones J.L., Coles A.J. (2014). Mode of action and clinical studies with alemtuzumab. Exp. Neurol..

[98] Guarnera C., Bramanti P., Mazzon E. (2017). Alemtuzumab: A review of efficacy and risks in the treatment of relapsing remitting multiple sclerosis. Ther. Clin. Risk Manag..

[99] De Weers M., Tai Y.T., van der Veer M.S., Bakker J.M., Vink T., Jacobs D.C., Oomen L.A., Peipp M., Valerius T., Slootstra J.W. (2011). Daratumumab, a novel therapeutic human CD38 monoclonal antibody, induces killing of multiple myeloma and other hematological tumors. J. Immunol..

[100] Palumbo A., Chanan-Khan A., Weisel K., Nooka A.K., Masszi T., Beksac M., Spicka I., Hungria V., Munder M., Mateos M.V. (2016). Daratumumab, Bortezomib, and Dexamethasone for Multiple Myeloma. N. Engl. J. Med..

[101] Campbell K.S., Cohen A.D., Pazina T. (2018). Mechanisms of NK Cell Activation and Clinical Activity of the Therapeutic SLAMF7 Antibody, Elotuzumab in Multiple Myeloma. Front. Immunol..

[102] Trudel S., Moreau P., Touzeau C. (2019). Update on elotuzumab for the treatment of relapsed/refractory multiple myeloma: Patients’ selection and perspective. OncoTargets Ther..

[103] Molhoj M., Crommer S., Brischwein K., Rau D., Sriskandarajah M., Hoffmann P., Kufer P., Hofmeister R., Baeuerle P.A. (2007). CD19-/CD3-bispecific antibody of the BiTE class is far superior to tandem diabody with respect to redirected tumor cell lysis. Mol. Immunol..

[104] Lee K.J., Chow V., Weissman A., Tulpule S., Aldoss I., Akhtari M. (2016). Clinical use of blinatumomab for B-cell acute lymphoblastic leukemia in adults. Ther. Clin. Risk Manag..

[105] Witzig T.E., Flinn I.W., Gordon L.I., Emmanouilides C., Czuczman M.S., Saleh M.N., Cripe L., Wiseman G., Olejnik T., Multani P.S. (2002). Treatment with ibritumomab tiuxetan radioimmunotherapy in patients with rituximab-refractory follicular non-Hodgkin’s lymphoma. J. Clin. Oncol..

[106] Sachpekidis C., Jackson D.B., Soldatos T.G. (2019). Radioimmunotherapy in Non-Hodgkin’s Lymphoma: Retrospective Adverse Event Profiling of Zevalin and Bexxar. Pharmaceuticals.

[107] Morschhauser F., Dekyndt B., Baillet C., Barthelemy C., Malek E., Fulcrand J., Bigot P., Huglo D., Decaudin B., Simon N. (2018). A new pharmacokinetic model for (90)Y-ibritumomab tiuxetan based on 3-dimensional dosimetry. Sci. Rep..

[108] Van de Donk N.W., Dhimolea E. (2012). Brentuximab vedotin. mAbs.

[109] Jalan P., Mahajan A., Pandav V., Bekker S., Koirala J. (2012). Brentuximab associated progressive multifocal leukoencephalopathy. Clin. Neurol. Neurosurg..

[110] Oak E., Bartlett N.L. (2016). A safety evaluation of brentuximab vedotin for the treatment of Hodgkin lymphoma. Expert Opin. Drug Saf..

[111] McCann S., Akilov O.E., Geskin L. (2012). Adverse effects of denileukin diftitox and their management in patients with cutaneous T-cell lymphoma. Clin. J. Oncol. Nurs..

[112] Yalici-Armagan B., Ayanoglu B.T., Demirdag H.G. (2019). Targeted tumour therapy induced papulopustular rash and other dermatologic side effects: A retrospective study. Cutan. Ocul. Toxicol..

[113] Beech J., Germetaki T., Judge M., Paton N., Collins J., Garbutt A., Braun M., Fenwick J., Saunders M.P. (2018). Management and grading of EGFR inhibitor-induced cutaneous toxicity. Future Oncol..

[114] Morano F., Corallo S., Lonardi S., Raimondi A., Cremolini C., Rimassa L., Murialdo R., Zaniboni A., Sartore-Bianchi A., Tomasello G. (2019). Negative Hyperselection of Patients With RAS and BRAF Wild-Type Metastatic Colorectal Cancer Who Received Panitumumab-Based Maintenance Therapy. J. Clin. Oncol..

[115] Han S.S., Lee M., Park G.H., Bang S.H., Kang Y.K., Kim T.W., Lee J.L., Chang H.M., Ryu M.H. (2010). Investigation of papulopustular eruptions caused by cetuximab treatment shows altered differentiation markers and increases in inflammatory cytokines. Br. J. Dermatol..

[116] Cardinale D., Ciceri F., Latini R., Franzosi M.G., Sandri M.T., Civelli M., Cucchi G., Menatti E., Mangiavacchi M., Cavina R. (2018). Anthracycline-induced cardiotoxicity: A multicenter randomised trial comparing two strategies for guiding prevention with enalapril: The International CardioOncology Society-one trial. Eur. J. Cancer.

[117] Bose P., Vachhani P., Cortes J.E. (2017). Treatment of Relapsed/Refractory Acute Myeloid Leukemia. Curr. Treat. Options Oncol..

[118] Iqubal A., Iqubal M.K., Sharma S., Ansari M.A., Najmi A.K., Ali S.M., Ali J., Haque S.E. (2019). Molecular mechanism involved in cyclophosphamide-induced cardiotoxicity: Old drug with a new vision. Life Sci..

[119] Adao R., de Keulenaer G., Leite-Moreira A., Bras-Silva C. (2013). Cardiotoxicity associated with cancer therapy: Pathophysiology and prevention strategies. Rev. Port. Cardiol..

[120] Litzow M.R. (2008). Arsenic trioxide. Expert Opin. Pharmacother..

[121] Hochhaus A., Larson R.A., Guilhot F., Radich J.P., Branford S., Hughes T.P., Baccarani M., Deininger M.W., Cervantes F., Fujihara S. (2017). Long-Term Outcomes of Imatinib Treatment for Chronic Myeloid Leukemia. N. Engl. J. Med..

[122] Curigliano G., Cardinale D., Dent S., Criscitiello C., Aseyev O., Lenihan D., Cipolla C.M. (2016). Cardiotoxicity of anticancer treatments: Epidemiology, detection, and management. CA Cancer J. Clin..

[123] Bretagne M., Lebrun-Vignes B., Pariente A., Shaffer C.M., Malouf G.G., Dureau P., Potey C., Funck-Brentano C., Roden D.M., Moslehi J.J. (2020). Heart failure and atrial tachyarrhythmia on abiraterone: A pharmacovigilance study. Arch. Cardiovasc. Dis..

[124] Holthof L.C., Mutis T. (2020). Challenges for Immunotherapy in Multiple Myeloma: Bone Marrow Microenvironment-Mediated Immune Suppression and Immune Resistance. Cancers.

[125] Turner N.C., Slamon D.J., Ro J., Bondarenko I., Im S.A., Masuda N., Colleoni M., DeMichele A., Loi S., Verma S. (2018). Overall Survival with Palbociclib and Fulvestrant in Advanced Breast Cancer. N. Engl. J. Med..

[126] Ramchand S.K., Cheung Y.M., Yeo B., Grossmann M. (2019). The effects of adjuvant endocrine therapy on bone health in women with breast cancer. J. Endocrinol..

[127] Qin Y., Qin Z.D., Chen J., Cai C.G., Li L., Feng L.Y., Wang Z., Duns G.J., He N.Y., Chen Z.S. (2019). From Antimicrobial to Anticancer Peptides: The Transformation of Peptides. Recent Pat. Anticancer Drug Discov..

[128] Solomon D.H., Glynn R.J., Karlson E.W., Lu F., Corrigan C., Colls J., Xu C., MacFadyen J., Barbhaiya M., Berliner N. (2020). Adverse Effects of Low-Dose Methotrexate: A Randomized Trial. Ann. Intern. Med..

[129] Saini L., Brandwein J. (2017). New Treatment Strategies for Philadelphia Chromosome-Positive Acute Lymphoblastic Leukemia. Curr. Hematol. Malig. Rep..

[130] Henningsson A., Karlsson M.O., Vigano L., Gianni L., Verweij J., Sparreboom A. (2001). Mechanism-based pharmacokinetic model for paclitaxel. J. Clin. Oncol..

[131] Inoue K., Ninomiya J., Saito T., Okubo K., Nakakuma T., Yamada H., Kimizuka K., Higuchi T., SBCCSG-36 Investigators (2019). Eribulin, trastuzumab, and pertuzumab as first-line therapy for patients with HER2-positive metastatic breast cancer: A phase II, multicenter, collaborative, open-label, single-arm clinical trial. Invest. New Drugs.

[132] Van Ramshorst M.S., van der Voort A., van Werkhoven E.D., Mandjes I.A., Kemper I., Dezentje V.O., Oving I.M., Honkoop A.H., Tick L.W., van de Wouw A.J. (2018). Neoadjuvant chemotherapy with or without anthracyclines in the presence of dual HER2 blockade for HER2-positive breast cancer (TRAIN-2): A multicentre, open-label, randomised, phase 3 trial. Lancet Oncol..

[133] Neven P., Jongen L., Lintermans A., Van Asten K., Blomme C., Lambrechts D., Poppe A., Wildiers H., Dieudonne A.S., Brouckaert O. (2018). Tamoxifen Metabolism and Efficacy in Breast Cancer: A Prospective Multicenter Trial. Clin. Cancer Res..

[134] Oprea A.D. (2017). Chemotherapy Agents With Known Pulmonary Side Effects and Their Anesthetic and Critical Care Implications. J. Cardiothorac. Vasc. Anesth..

[135] Petrelli F., Ardito R., Ghidini A., Zaniboni A., Ghidini M., Barni S., Tomasello G. (2018). Different Toxicity of Cetuximab and Panitumumab in Metastatic Colorectal Cancer Treatment: A Systematic Review and Meta-Analysis. Oncology.

[136] Almazroo O.A., Miah M.K., Venkataramanan R. (2017). Drug Metabolism in the Liver. Clin. Liver Dis..

[137] Jain T., Litzow M.R. (2020). Management of toxicities associated with novel immunotherapy agents in acute lymphoblastic leukemia. Ther. Adv. Hematol..

[138] Han J.M., Yee J., Cho Y.S., Gwak H.S. (2020). Factors Influencing Imatinib-Induced Hepatotoxicity. Cancer Res. Treat..

[139] Sahni V., Choudhury D., Ahmed Z. (2009). Chemotherapy-associated renal dysfunction. Nat. Rev. Nephrol..

[140] Kabra R., Singh S. (2021). Transporter proteins and its implication in human diseases. Adv. Protein Chem. Struct. Biol..

[141] Joyce H., McCann A., Clynes M., Larkin A. (2015). Influence of multidrug resistance and drug transport proteins on chemotherapy drug metabolism. Expert Opin. Drug Metab. Toxicol..

[142] Liu X. (2019). Transporter-Mediated Drug-Drug Interactions and Their Significance. Adv. Exp. Med. Biol..

[143] Xiao H., Zheng Y., Ma L., Tian L., Sun Q. (2021). Clinically-Relevant ABC Transporter for Anti-Cancer Drug Resistance. Front. Pharmacol..

[144] Olusanya T.O.B., Haj Ahmad R.R., Ibegbu D.M., Smith J.R., Elkordy A.A. (2018). Liposomal Drug Delivery Systems and Anticancer Drugs. Molecules.

[145] Renton K.W. (2005). Regulation of drug metabolism and disposition during inflammation and infection. Expert Opin. Drug Metab. Toxicol..

[146] Scripture C.D., Figg W.D. (2006). Drug interactions in cancer therapy. Nat. Rev. Cancer.

[147] Bellmann R., Smuszkiewicz P. (2017). Pharmacokinetics of antifungal drugs: Practical implications for optimized treatment of patients. Infection.

[148] Deb S., Pandey M., Adomat H., Guns E.S. (2012). Cytochrome P450 3A-mediated microsomal biotransformation of 1alpha,25-dihydroxyvitamin D3 in mouse and human liver: Drug-related induction and inhibition of catabolism. Drug Metab. Dispos..

[149] Manikandan P., Nagini S. (2018). Cytochrome P450 Structure, Function and Clinical Significance: A Review. Curr. Drug Targets.

[150] Mittal B., Tulsyan S., Kumar S., Mittal R.D., Agarwal G. (2015). Cytochrome P450 in Cancer Susceptibility and Treatment. Adv. Clin. Chem..

[151] Harmsen S., Meijerman I., Beijnen J.H., Schellens J.H. (2007). The role of nuclear receptors in pharmacokinetic drug-drug interactions in oncology. Cancer Treat. Rev..

[152] Tremaine L., Brian W., DelMonte T., Francke S., Groenen P., Johnson K., Li L., Pearson K., Marshall J.C. (2015). The role of ADME pharmacogenomics in early clinical trials: Perspective of the Industry Pharmacogenomics Working Group (I-PWG). Pharmacogenomics.

[153] Huang T., Song X., Yang Y., Wan X., Alvarez A.A., Sastry N., Feng H., Hu B., Cheng S.Y. (2018). Autophagy and Hallmarks of Cancer. Crit. Rev. Oncog..

[154] Lolodi O., Wang Y.M., Wright W.C., Chen T. (2017). Differential Regulation of CYP3A4 and CYP3A5 and its Implication in Drug Discovery. Curr. Drug Metab..

[155] Chan C.W.H., Law B.M.H., So W.K.W., Chow K.M., Waye M.M.Y. (2020). Pharmacogenomics of breast cancer: Highlighting CYP2D6 and tamoxifen. J. Cancer Res. Clin. Oncol..

[156] Lam S.W., Guchelaar H.J., Boven E. (2016). The role of pharmacogenetics in capecitabine efficacy and toxicity. Cancer Treat. Rev..

[157] Robey R.W., Pluchino K.M., Hall M.D., Fojo A.T., Bates S.E., Gottesman M.M. (2018). Revisiting the role of ABC transporters in multidrug-resistant cancer. Nat. Rev. Cancer.

[158] Song W., Li D., Tao L., Luo Q., Chen L. (2020). Solute carrier transporters: The metabolic gatekeepers of immune cells. Acta Pharm. Sin. B.

[159] Wilson N.C., Choudhury A., Carstens N., Mavri-Damelin D. (2017). Organic Cation Transporter 2 (OCT2/SLC22A2) Gene Variation in the South African Bantu-Speaking Population and Functional Promoter Variants. OMICS J. Integr. Biol..

[160] Chakraborty S., Hosen M.I., Ahmed M., Shekhar H.U. (2018). Onco-Multi-OMICS Approach: A New Frontier in Cancer Research. BioMed Res. Int..

[161] Li W., Pang I.H., Pacheco M.T.F., Tian H. (2018). Ligandomics: A paradigm shift in biological drug discovery. Drug Discov. Today.

[162] Hasin Y., Seldin M., Lusis A. (2017). Multi-omics approaches to disease. Genome Biol..

